# A systematic review protocol: health economic evaluations of immunotherapy and biologics for food allergy management

**DOI:** 10.1186/s13223-024-00909-4

**Published:** 2024-09-19

**Authors:** Andrew T. Fong, Joshua Jacob, Jennifer L. P. Protudjer, Melanie Lloyd, Liz Thyer, Peter S. Hsu, Lei Si

**Affiliations:** 1 School of Health Sciences, Western Sydney University, Campbelltown, Australia; 2 The Children’s Hospital at Westmead, Westmead, NSW Australia; 3 School of Medicine, Western Sydney University, Campbelltown, Australia; 4 Campbelltown Hospital, Campbelltown, NSW Australia; 5 Department of Pediatrics and Child Health, Rady Faculty of Health Sciences, Max Rady College of Medicine, University of Manitoba, Winnipeg, MB Canada; 6 Children’s Hospital Research Institute of Manitoba, Winnipeg, MB Canada; 7 Department of Food and Human Nutritional Sciences, Faculty of Agricultural and Food Sciences, University of Manitoba, Winnipeg, MB Canada; 8 George and Fay Yee Centre for Healthcare Innovation, Winnipeg, MB Canada; 9 Institute of Environmental Medicine, Karolinska Institutet, Stockholm, Sweden; 10 Monash University, Melbourne, Australia; 11 Murdoch Children’s Research Institute, Melbourne, Australia

To the Editor,

Food allergy is a significant public health concern, that currently affects an estimated 4–10% of people worldwide and the prevalence is thought to be increasing [1]. Food allergy typically demands that those affected avoid consumption of known allergens, which contributes to requisite dietary and behavioural changes. In turn, such changes and the potential for severe reactions, contributes to substantial economic costs, including the cost of treatment, healthcare service utilisation, carers’ time and so on. These economic costs of food allergy are spread throughout families, communities, and society at large. With novel approaches to therapies and evolving discussions on allergy management, there are multiple knowledge gaps on the economic burden of food allergies and the cost-effectiveness of the various treatments available [2].

Over the last couple of years, food allergen immunotherapy has shown promise in the management of food allergies, reducing the incidence of serious allergic reactions and potentially inducing sustained unresponsiveness (remission of food allergy) in a subset of patients [3, 4]. Commerical forms of food allergen immunotherapy are now available in some countries with additional products in the pipeline. In addition, multiple protocols for clinician-led immunotherapy have been proposed. Despite this, the economic viability and cost-effectiveness of these products have been varied [5]. A previous systematic review broadly exploring the cost-effectiveness of food allergy interventions in children, identified only three articles related to immunotherapy with no studies identified on biologics. However, this is an area with increased interest and a timely update is warranted due to the rapidly evolving evidence base [5].

For biological therapies, Wood et al. (2024) recently highlighted that a well-established monoclonal anti-Immunoglobulin E (IgE) antibody, Omalizumab, has clinical efficacy compared to a placebo in increasing the reaction threshold for peanut and other common food allergens in children and adults [6]. The varying models of care for administration and monitoring in international health systems add difficulty for assessment of cost-effectiveness for this novel food allergy treatment modality.

We will undertake a systematic review with the aim to synthesise health economic evaluation studies on the use of immunotherapy and biologics in food allergy management.

This systematic review protocol was developed based on the Preferred Reporting Items for Systematic Reviews and Meta-Analyses (PRISMA) statement published and updated in 2020 [7]. A PRISMA checklist and PRISMA flow diagram will be completed with reporting. The review is registered (registration number: CRD42024531663) on the International Prospective Register of Systematic Reviews (PROSPERO) prior to commencement.

Three routes of food allergen immunotherapy will be included in the review: oral immunotherapy (OIT), sublingual immunotherapy (SLIT) and epicutaneous immunotherapy (EPIT). Food allergen immunotherapy typically requires long-term ongoing daily exposure to the culprit allergen, with the aim of increasing the food allergen reaction threshold (termed “desensitisation”), and decreasing severe allergic reactions. Some studies have also explored the ability of food immunotherapy to induce “sustained unresponsiveness” (SU), where the allergic response remains suppressed even after a period of dietary exclusion of the culprit allergen. SU is considered to be a superior outcome to desensitisation because it permits the patient to cease daily dosing and consume the allergen freely as part of their regular diet. Biologics used for the treatment of food allergies including (but not limited to) Omalizumab, a monoclonal anti-IgE antibody and Dupilumab, a monoclonal antibody directed towards the interleukin-4 (IL-4) and interleukin-13 cytokines (IL-13) will be included in this review. These biologic agents have established uses in a range of atopic diseases through the blockade of immunological and allergic pathways and are administered as subcutaneous injections every 2–4 weeks.

A comprehensive literature search will be conducted using a highly sensitive search strategy (DOI: 10.1079/searchrxiv.2024.00594) applied to databases including Medline (OVID), Embase (OVID), Cochrane Library, Health Technology Assessment (HTA), NHS Economic Evaluation Database (EED), EconLit and SCOPUS. Bibliographies of eligible publications will be reviewed for suitable articles and subsequently included. ‘Grey’ literature will be reviewed including cost-effectiveness studies and economic evaluations not published through peer-reviewed journals. This will include websites and databases such as the Pharmaceutical Benefits Scheme (PBS) in Australia, CEA Registry, Canadian Agency for Drugs and Technologies in Health (CADTH), Institute for Clinical and Economic Review (IER), and the National Institute for Health and Care Excellence (NICE) in the UK. Official websites and research publications related to commercially available products identified will also be hand searched for economic evaluation data.

Economic evaluation studies of immunotherapy or biologic food allergy treatments in humans published will be included. These studies could include cost-effectiveness analysis, cost-benefit analysis, cost-utility analysis and cost-minimization analysis. There will be no restriction of publication date. Articles that do include original data such as reviews, commentaries, perspectives, conference abstracts or editorials will be excluded. Cost comparison, cost descriptions, cost outcome descriptions, and cost-of-illness studies will be excluded. Articles published in languages other than English will be excluded.

The retrieved citations will be uploaded to Covidence (Veritas Health Innovation, Melbourne, Victoria, Australia) after the initial search. Following deduplication, two reviewers (AF and JJ) will independently conduct title and abstract screening of the remaining studies in a blinded fashion. Following title and abstract screening, full texts of the selected studies will be uploaded to Covidence. Independent screening of the full texts will similarly occur with the results then unblinded. In the event of conflicts regarding the inclusion of an article, the screeners will convene a meeting to discuss the discrepancies with consultation of a third reviewer (LS).

For all included articles, relevant data including study setting, design, study period, population, intervention, comparator, method of economic evaluation, economic perspective, type of costs included, results, and conclusions will be extracted and organized into tables. Cost-effectiveness measures, including cost per quality-adjusted life year (QALY), incremental cost-effectiveness ratio (ICER), net health benefit, net monetary benefit, return on investment, and cost benefit ratio will be recorded. The methodological quality of the included studies will be assessed using the Consolidated Health Economic Evaluation Reporting Standards (CHEERS) checklist [8] and the Philips Checklist [9].

Extracted data from the articles will be used to provide a detailed narrative synthesis of studies. As significant heterogeneity in populations, interventions, comparators, methodology and reporting outcomes is expected, a meta-analysis is not planned. Furthermore, the results will highlight the strengths and limitations of the included studies and the health economic evaluations assessed. We anticipate that findings from the current review will not only help clinicians make an informed choice when prescribing immunotherapy and biologics but also highlight gaps, limitations and areas of improvement for existing models of care.

## Data Availability

No datasets were generated or analysed during the current study.

## Abbreviations

IgE: Immunoglobulin E
SU: Sustained unresponsiveness
OIT: Oral immunotherapy
SLIT: Sublingual Immunotherapy
EPIT: Epicutaneous immunotherapy
IL: 4–interleukin–4
IL: 13–interleukin–13
QALY: Quality–adjusted life year
ICER: Incremental cost–effectiveness ratio
